# Micro- and Nanoengineering Approaches to Control Stem Cell-Biomaterial Interactions

**DOI:** 10.3390/jfb2030088

**Published:** 2011-06-24

**Authors:** Alireza Dolatshahi-Pirouz, Mehdi Nikkhah, Kristian Kolind, Mehmet R. Dokmeci, Ali Khademhosseini

**Affiliations:** 1 Center for Biomedical Engineering, Department of Medicine, Brigham and Women's Hospital,Harvard Medical School, Boston, MA 02139, USA; E-Mails: adolatshahi-pirouz@rics.bwh.harvard.edu (A.D.-P.); mnikkhah@rics.bwh.harvard.edu (M.N.); mehmetd@mit.edu (M.R.D.); 2 Harvard-MIT Division of Health Sciences and Technology, Massachusetts Institute of Technology,Cambridge, MA 02139, USA; 3 Interdisciplinary Nanoscience Center (iNANO), Aarhus University, DK-8000 Aarhus C, Denmark;E-Mail: kristian.kolind@inano.au.dk; 4 Wyss Institute for Biologically Inspired Engineering, Harvard University, Cambridge,MA 02139, USA

**Keywords:** micro- and nanotopography, microwells, microarrays, embryonic and adult stem cells, stem cell therapy

## Abstract

As our population ages, there is a greater need for a suitable supply of engineered tissues to address a range of debilitating ailments. Stem cell based therapies are envisioned to meet this emerging need. Despite significant progress in controlling stem cell differentiation, it is still difficult to engineer human tissue constructs for transplantation. Recent advances in micro- and nanofabrication techniques have enabled the design of more biomimetic biomaterials that may be used to direct the fate of stem cells. These biomaterials could have a significant impact on the next generation of stem cell based therapies. Here, we highlight the recent progress made by micro- and nanoengineering techniques in the biomaterials field in the context of directing stem cell differentiation. Particular attention is given to the effect of surface topography, chemistry, mechanics and micro- and nanopatterns on the differentiation of embryonic, mesenchymal and neural stem cells.

## 1. Introduction

With the increasing number of patients suffering from damaged or diseased organs and the shortage of organ donors, the need for methods to construct human tissues outside the body has risen. To address this issue, the interdisciplinary field of tissue engineering has emerged in the past few years to generate biological tissue constructs that maintain or enhance normal tissue function [1,2].

One of the current challenges in the development of tissue engineered constructs is the lack of a renewable cell source. Embryonic, induced pluripotent and adult stem cells are promising cell sources in therapeutic and regenerative medicine. Due to their ability to self-renew and differentiate into various cell types, these cells could potentially be cultured and harvested for regeneration of damaged, injured and aged tissue [3,4]. Embryonic stem cells (ESC) are pluripotent with the ability to differentiate into cells of all three germ layers, ectoderm, endoderm, and mesoderm, whereas adult stem cells (ASC) are multipotent with the capacity to differentiate into a limited number of cell types [5]. For instance, mesenchymal stem cells (MSCs) which reside in the bone marrow, can differentiate into bone (osteoblasts) [6], muscle (myoblasts) [7], fat (adipocytes) [8] and cartilage (chrondocytes) [5] cells, while neural stem cells (NSCs) either give rise to support cells in the nervous system of vertebrates (astrocytes and oligodendrocytes) or neurons [9].

*In vivo*, differentiation and self-renewal of stem cells is dominated by signals from their surrounding microenvironment [10]. This microenvironment or “niche” is composed of other cell types as well as numerous chemical, mechanical and topographical cues at the micro- and nanoscale, which are believed to serve as signaling mechanisms to control the cell behavior [11]. For instance, extracellular matrix (ECM) molecules such as collagen [12] as well as the basement membrane of the tissue matrix [13] contain micro- and nanoscale features. Tissue stiffness is also known to vary depending on the organ type, disease state and aging process [14,15,16]. In tissue culture, stem cell differentiation has traditionally been controlled by the addition of soluble factors to the growth media [17]. However, despite much research, most stem cell differentiation protocols yield heterogeneous cell types [18,19]. Therefore, it is desirable to use more biomimetic *in vitro* culture conditions to regulate stem cell differentiation and self-renewal.

Recent advances in micro- and nanofabrication technology have paved the way to create substrates with precise micro- and nanocues, variable stiffness and chemical composition to better mimic the *in vivo* microenvironment [2,20,21]. By employing approaches such as self-assembled monolayers (SAMs), microcontact printing, e-beam, photo- and soft lithography, tissue engineers aim to incorporate topographical, mechanical and chemical cues into biomaterials to control stem cell fate decisions [2,21,22]. This review highlights recent progress made by using micro- and nanoengineered biomaterials to direct the fate of stem cells, with particular emphasis on ESCs, MSCs and NSCs.

## 2. Biomaterials with Micro- and Nanoscale Features for Directing Stem Cell Fate

### 2.1. Stem Cell Niche in Vivo

**Figure 1:**
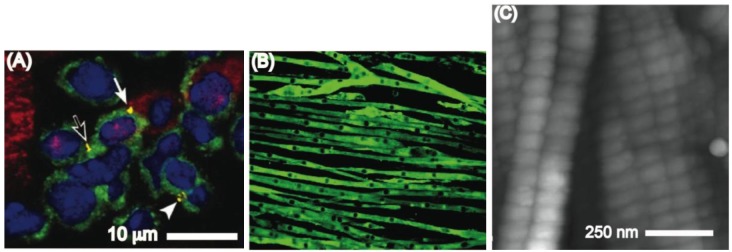
(**A**) Cluster of cardiac stem cells (green) and lineage committed cells (red). The yellow regions stain for connexin 43 (Published with permission from PNAS [24]); (**B**) A fluorescent image in which myoblast actin filaments are stained green and the myoblasts nuclei are shown as dark elongated spots. Each myoblast tube is measured to be approximately 12.5 μm in diameter (Published with permission from Am Physiol Soc [25]); (**C**) Atomic force microscopy images of the D-band patterns on collagen I (Published with permission from Royal society Publishing [27]).

In the body the cellular microenvironment is comprised of other cells, matrix, and soluble factors that regulate the resulting cell behavior [22,23]. Direct cell-cell contact is an important regulator of cellular processes as well as tissue architecture. For instance, cell-cell contacts regulate the cardiac stem cell microenvironment (Figure 1A) and direct mature cardiomyocytes to form fibrous microstructures (Figure 1A) [24]. Furthermore, during myogenesis, myoblasts assemble into microscale tubes (Figure 1B) [25]. Nanotopographies in the basement membrane also affect cells [26]. These topographies are mainly composed of networks of nanoscale pores, ridges, and fibers made by ECM molecules such as collagen, fibronectin and laminin [26]. In addition, hydroxyapatite crystals and cell adhesive proteins such as osteopontin, osteocalcin and fibronectin can bind to collagen fibers (Figure 1C) [26,27] resulting in discrete nanopatterns of cell adhesive and mineral patches [26]. In summary, cells encounter and respond to topography in the *in vivo* environment at length scales ranging from the nano- to microscale [26]. It is therefore important to incorporate features at such length scales into the development of biomaterial-based platforms suitable for stem cell therapies.

### 2.2. Stem Cell Interactions with Microtopographies

**Figure 2:**
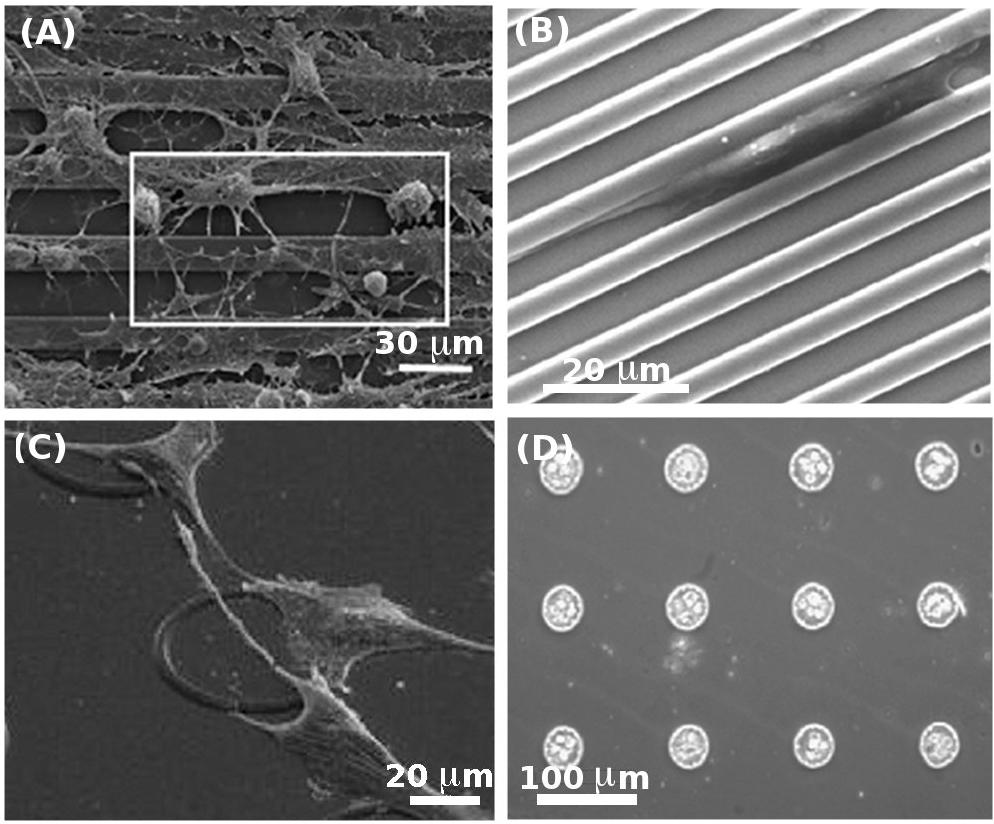
(**A**) Co-culture of adult rat hippocampal progenitor cells (AHPCs) with astrocytes on microgrooved PS substrate. The square illustrates how the cells align in the same direction as the microgrooves (Published with permission from Elsevier [45]); (**B**) Elongation of mouse mesenchymal stem cells (mMSCs) inside microgrooves on a silicon substrate (Published with permission from Elsevier [46]); (**C**) Attachment of rMSCs on ring shaped PMMA microstructures (Published with permissions from Elsevier [48]); (**D**) Formation of embryoid bodies (EBs) using an array of PEG microwells (Published with permission from Elsevier [53]).

With the advances in photo- and soft lithographic techniques, there has been a growing interest towards fabrication of micro- and nanotopographies to address fundamental questions related to cell-substrate interactions. An excellent example is the alignment of cells along microgrooves, a phenomenon known as contact guidance [28,29,30,31]. Microstructures also influence basic cellular processes such as adhesion [32,33,34,35,36,37], migration [38,39,40], proliferation [41,42] and differentiation [43,44]. Recently, there has been significant interest towards utilizing microscale topographies in controlling stem cell behavior. Most of these studies have examined the effect of microgrooved topographies on alignment, morphology and differentiation of stem cells. In a study by Mallapragada *et al.* [45], adult rat hippocampal progenitor cells (AHPCs) exhibited an elongated morphology along microgrooved laminin coated polystyrene (PS) substrates (Figure 2A). On these substrates, the elongated morphology of the cells remained intact after seeding cortical astrocytes with the AHPCs, and the differentiation of AHPCs towards an early neural phenotype (III β-tubulin) was enhanced after co-culturing. Likewise, mouse mesenchymal stem cells (mMSCs) were shown to exhibit an elongated morphology when cultured on microgrooves [46] (Figure 2B). A number of studies have investigated the effect of microgroove widths on differentiation of MSCs [44,47]. It was shown that the differentiation of MSCs into neural-like cells was less pronounced on the 4 μm wide microgrooves compared to narrower 1 and 2 μm microgrooves. In addition, MSCs on 1 μm and 2 μm grooves showed an upregulation of the expression neurogenic markers such as microtubule associate protein 2 (MAP2) and neural nuclei (NeuN) [44]. In another study, Kurpinski *et al.* [47] applied a uniaxial strain to an elastomeric PDMS substrate containing parallel microgrooves on its surface. The uniaxial mechanical stimuli resulted in stem cell alignment along the microgrooves and an increase in cell proliferation. It was also evident that there was an increased expression of calponin 1, a gene-marker of smooth muscle cell contractility after 2 and 4 days of culture under induced mechanical strain.

The shape of the microtopographies has also been shown to be important in stem cell behavior [48,49]. For example Engel *et al.*, [48] developed ring and square shaped poly(methyl methacrylate) (PMMA) patterns to control the attachment of rat MSCs (rMSCs) (Figure 2C). They showed that the attachment of rMSCs was most favorable to ring shaped microstructures compared to other geometries, while cell proliferation and differentiation were the same on the microstructured and flat surfaces. In another study [49], hMSCs were cultured on concave and convex shaped poly(L-Lactic-Acid) (PLLA) microtopographies. More than 50% of cells expressed CD71 after 10 days on the concave and convex surfaces confirming that hMSCs maintained their proliferative ability. Additionally the authors observed enhanced cell spreading on concave surfaces compared to the convex ones.

Non-adhesive microscale structures also direct cell differentiation. For example, it is widely recognized that the differentiation of ESCs into various cell types could benefit from cell aggregates called embryoid bodies (EBs) [50]. Typically, EBs are generated in non-adhesive dishes to yield cell aggregates of various sizes. To generate more homogenous EBs, the hanging drop method is used, however this technique is cumbersome and difficult to scale-up [51]. Recently, microtopography has been employed to generate homogenously sized EBs by trapping cells inside microwells with different diameters [52]. For example, non-adhesive PEG microwells have been used to generate and retrieve EBs of controlled sizes [53]. Such microwells have also been shown to direct the differentiation of stem cells by modulating the size of EBs [43]. In particular, larger EBs (450 μm) resulted in more cardiac cells whereas smaller EBs (150 μm) generated more endothelial cells. This behavior was shown to be regulated by the differential expression of non-canonical Wnt pathway molecules, Wnt5a and Wnt11. Overall, the above-mentioned studies demonstrated the potential of microstructures for creating EBs with a homogenous size distribution and in directing the fate of ESCs.

### 2.3. Stem Cell Behavior on Micropatterned Surfaces

Micropatterned substrates have been used extensively to pattern cells on substrates and to control the resulting cell shape [8,54,55,56]. These studies have revealed that cell shape is an important regulator of apoptosis [54,56], proliferation and differentiation [55]. For instance, McBeath *et al.* [55] demonstrated that hMSCs that were spread on large protein patterns differentiated into osteogenic cells, while rounded cells on smaller patterns generated adipogenic cells. In another study, Kilian *et al.* [8] explored the differentiation of hMSCs into osteogenic and adipogenic cells on different micropatterns. It was shown that cell attachment on ellipsoid and star shaped fibronectin micropatterns enhanced the differentiation into bone cells compared to square shaped geometries.

Micropatterns have also been used to demonstrate a relationship between NSCs shape and differentiation. Solanki *et al.* [9] was able to control the fate of rat NSCs (rNSCs) by varying the geometry and dimensions (10–250 μm) of laminin patterns. They reported that grid patterns resulted in axon-like outgrowths from the cell body accompanied by neural differentiation, while square shaped islands resulted in an increase in the number of cells expressing astrocyte markers. Likewise, a more stellate-like cell morphology was observed by Ruiz *et al.* [57] on grid shaped micropatterns compared to a nonpatterned surface. The stellate morphology resulted in an enhanced expression of the neural marker β-TubIII. In summary, these reports [8,9,57] validate the feasibility of controlling the fate of both MSCs and NSCs by culturing cells on micropatterned surfaces.

### 2.4. Nanoscale Engineering Approaches for Controlling Stem Cell Fate

Early studies of cells on nanostructured surfaces have mainly focused on nanogrooves. These studies have demonstrated that nanoscale grooves can direct cell alignment and migration through contact guidance even on feature sizes that were only 30 nm deep [58,59,60,61]. Moreover, studies have shown that stem cell alignment on nanogrooves lead to a more pronounced differentiation profile. In the paper by Lee *et al.*, polymeric nanogrooves (350 nm wide) were used to demonstrate a correlation between cell alignment and hESCs differentiation into the neuronal linage [62]. A similar relationship between the alignment of hMSCs and their neuronal differentiation was shown using 350 nm wide grooves by Yim *et al.* [63]. With the advances in the nanofabrication technology, other topographies have now become available for use in stem cell studies. For example, approaches such as polymer phase separation [64,65,66], metal anodization [67,68,69], dip-pen nanolithography [70], colloidal lithography [71,72,73], UV-assisted capillary force lithography [62,74,75] molecular beam epitaxy (MBE) [76,77,78], and glancing angle deposition [79,80] have been employed to fabricate sub-100 nm nanotubes, islands, and pyramids.

**Figure 3:**
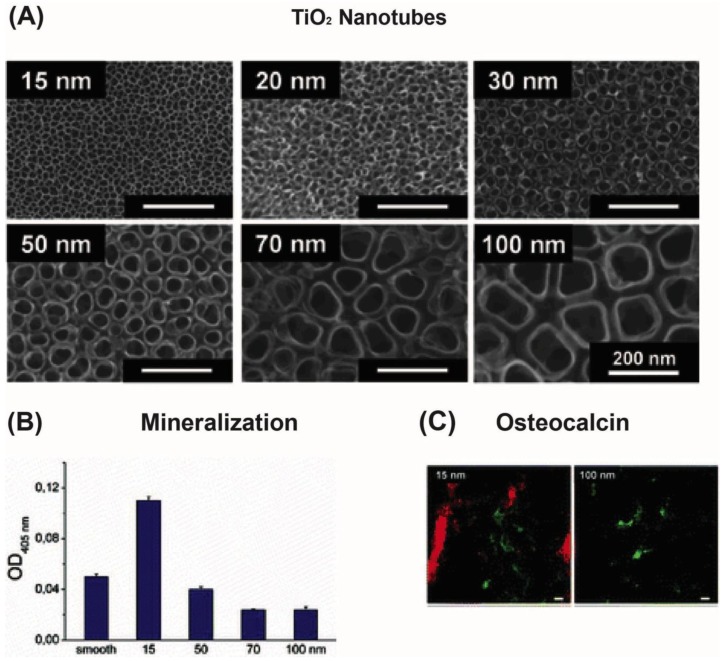
(**A**) Scanning electron micrographs of the TiO_2_-nanotubes (15, 20, 30, 50, 70, 100 nm); (**B**) Plot of alkaline phosphatase activity *versus* nanotube diameter; (**C**) Osteocalcin (red) and F-actin (green) staining of cells seeded on 15 nm and 100 nm TiO_2_-nanotubes. The scale bar is 20 μm (Published with permission from ACS publications [67]).

It has been shown that the nanostructured surfaces can direct MSCs into osteogenic cells [6,67,68,81,82,83]. Much of this focus has been on MSCs interaction with vertical TiO_2_ nanotubes fabricated by metal anodization [6,67,68,81,82]. Park *et al.* [67,68] performed extensive studies on the behavior of rMSCs on TiO_2_ nanotubes with tube diameters in the range of 15 to 100 nm (Figure 3A) [67,68]. A more pronounced cell response was observed on smaller nanotubes (15–30 nm), where cell adhesion, spreading, bone mineralization (Figure 3B) and bone marker expression (Figure 3C) were found to be enhanced compared to flat TiO_2_ [68,69,84]. Moreover, by examining the cytoskeletal structure of the cells, more focal contacts were observed on the smallest nanotubes [67], in agreement with the upregulated stem cell differentiation observed on the 15 and 30 nm nanotubes [85]. Other groups have observed similar behavior for hMSCs and rMSCs that were cultured on 70 and 100 nm TiO_2_ nanotubes [6,86]. For example, a higher alkaline phosphate activity was observed on 80 nm nanotubes followed by a larger mineralization of calcium and phosphate [86]. Furthermore, Oh *et al.* [6] found that the expression of bone proteins such as osteopontin and osteocalcin were significantly higher on 100 nm nanotubes. In conclusion, there is a strong indication that generation of nanotubes by metal anodization could enhance the performance of orthopedic titanium implants. This is either linked directly to mechanical stresses transmitted from the nanostructures to the cell nucleus [87,88,89] or indirectly by structural modulation of ECM proteins to expose cell adhesive domains [90,91,92] or a combination of both.

As described previously, the nanoscale chemical and topographical cues *in vivo* have different shapes and sizes. By employing UV-assisted capillary force lithography it is possible to examine stem cell behavior on nanotopographies with various chemistries, shapes and sizes [23,62,75]. In a study by You *et al.* [75], the osteogenic differentiation of hMSCs on polyurethane acrylate nanogrooves and columns with different sizes were investigated. They noticed that the highest expression of osteogenic markers and alkaline phosphatase activity was on the 400 nm wide nanocolumns.

Moreover, the distribution of topographical cues in the stem cell microenvironment may also influence stem cell behavior. Dalby *et al.* [83] found that surfaces composed of nanopits with controlled disorder resulted in increased expression of osteogenic markers relative to surfaces consisting of either highly ordered or randomly displaced nanopits. In another study by Hunt and coworkers [70], dip-pen nanolithography was used to fabricate nanopatterns with different chemistries and spacings for analysis of stem cell behavior [70]. Specifically, they patterned thiolated molecules terminated with various chemistries (including carboxyl, amino, methyl and hydroxyl) onto gold surfaces. The chemically functionalized islands were 70 nm wide with inter-island spacing that ranged from 140 to 1000 nm. They cultured hMSCs on the fabricated surfaces and found that the adhesion and expression of several stem cell markers depended on the specific chemistry and the distance between the nanoscale islands [70]. This approach provided an efficient method to precisely control size, spacing and chemistry of nanofabricated patterns and could in theory be used to fabricate randomly ordered nanoscale islands. Thus, various nanoscale fabrication methods can be used to create nanostructured surfaces for directing stem cell differentiation. These approaches are implemented in 2D, therefore to further advance the use of these systems to regulate stem cell behavior, it is necessary to implement the nanosculpturing in a 3D environment to better replicate the *in vivo* environment of the stem cells.

## 3. The Role of Chemical Moieties and Substrate Stiffness on Stem Cell Fate

### 3.1. Chemically Functionalized Surfaces

The stem cell microenvironment consists of numerous molecular cues including proteins and polysaccharides. It is becoming increasingly clear that the biochemical cues in the cellular microenvironment to a large extent determine processes such as cell attachment, proliferation and differentiation [93]. By using SAMs to coat surfaces, it is possible to test stem cell behavior on a range of chemistries [94,95]. Wu and coworkers [95] used SAMs with different chain lengths and hydrophobic head groups to develop various surface hydrophobicities. They observed that an increase in surface hydrophobicity resulted in higher hESC proliferation and differentiation [95]. In the future, such SAM coated surfaces could potentially be used to control cell size and enhance the differentiation profile of hESCs *in vitro*.

SAMS conjugated to various ligands, such as peptides or proteins, have also been synthesized and used in stem cell studies [96,97,98,99]. For example, surfaces functionalized with RGD ligands increased osteogenic [100,101,102,103], chondrogenic [104] and neurogenic [105] differentiation of stem cells compared to non-functionalized substrates. Although many studies have used RGD functionalized surfaces, different polymer coatings have likewise been used to induce stem cell differentiation. In one approach, Joy *et al.* [106] coated surfaces with different polymer compositions and observed that the surface chemistry had a significant influence on the osteogenic and adipogenic differentiation of hMSCs. However, after functionalization with RGD ligands, no differences were found in the expression of these markers between surfaces coated with different polymers. The fact that immobilized adhesive ligands can override the effect of the underlying polymer coating can have important implications in designing polymer-based biomaterials. In conclusion, altering the surface chemistry influences the behavior of stem cells and their differentiation in a notable way.

### 3.2. Substrate Stiffness

**Figure 4:**
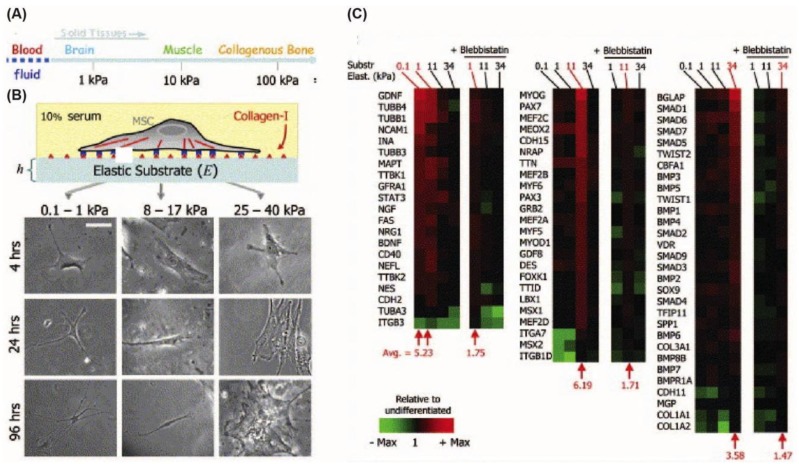
(**A**) The elastic moduli of different solid tissues ranging from blood to collagenous bone; (**B**) The images show how different substrate stiffness values influence cell morphology. Scale bar is 20 μm; (**C**) Microarray profiling of differentiation marker expression on substrates with different stiffnesses. The microarray profiling showed that neurogenic markers were highest on 0.1–1 kPa gels, while myogenic markers were highest on 11 kPa gels and osteogenic markers were highest on 34 kPa gels. (Published with permission from Elsevier [7]).

Mammalian cells can sense the elasticity of the substrates on which they are cultured [7]. This is caused by transmission of mechanical forces between substrate and cell, which generates contractile forces in the cell. These contractile forces in turn influence cell behaviors such as spreading [107,108], migration [109], proliferation [110] and apoptosis [111]. Pitelka and coworkers [112] provided early evidence in 1979 that substrate stiffness also affects differentiation. They found that mouse epithelial cells (mECs) differentiate better on softer collagen substrates compared to harder plastic tissue culture dishes. In another study, myoblasts were seeded on substrates with different stiffness [113] to show that actin/myosin striation, as it is seen in natural muscle, occurred only on the substrates with mechanical properties similar to that of a muscle [114]. More recently, Engler *et al.* [7] cultured hMSCs on a polyacrylamide gel homogeneously coated with collagen I ligands. The substrates had variable stiffness representing that of nerve (0.1–1 kPa), muscle (8–17 kPa) and bone tissue (25–40 kPa) and it was it was observed that the hMSCs differentiated along the neurogenic, myogenic and osteogenic lineage, respectively (Figure 4) [7]. Cooper-White and coworkers [113] further hypothesized that ECM proteins could influence hMSCs fate and therefore analyzed the combined effect of various ECM proteins (collagen I, collagen IV, laminin, and fibronectin). Their results revealed a significant interplay between ECM proteins and the underlying substrate elasticity affecting the myo- or osteogenic differentiation patterns. These studies suggest that both the elastic modulus of the substrate and the coated ECM proteins play a significant role in hMSC differentiation [7,113].

*In vivo*, stem cells exist in 3D microenvironments, hence it is important to understand the effect of 3D matrix stiffness on stem cell differentiation. Over the past few years, many new techniques have emerged to fabricate 3D constructs with precise mechanical properties [93]. In particular, hydrogels have proven as a promising tool for the fabrication of 3D microenvironments [115,116,117,118,119,120,121]. In one study Pek *et al.* [116] used a thixotropic polyethylene glycol-silica (PEG-silica) to generate 3D gels with different stiffnesses [116]. Their findings showed that the highest expression of neural (ENO2), myogenic (MYOG) and osteogenic (Runx2, OC) markers occurred on gels corresponding to low (7 Pa), intermediate (25 Pa) and high (75 Pa) gel stiffness respectively, consistent with previous findings on 2D surfaces [7].

### 3.3. High-Throughput Screening of Stem Cell Differentiation on Biomaterials

Most of today's biomaterials are prepared and tested individually for various applications. This process is time-consuming and expensive. An emerging approach in the development of biomaterials has been the use of combinatorial high-throughput screening methods to lower the cost and the experimentation time. This approach can be applied to stem cell bioengineering by simultaneously examining numerous parameters on stem cell fate. Kohn and co-workers [122] developed one of the first high-throughput biomaterial library systems in 1997. By employing polyacrylates, they successfully generated a microarray with 112 different combinations and used the array to examine fibroblast proliferation.

Microarray printing technologies have been more widely used in the biomaterials field over the past few years to screen for various stem cell material-interactions [100,123,124]. For example, Flaim *et al.* [124] used a DNA spotter to develop an ECM matrix microarray for probing the differentiation of primary rat hepatocyte cells (rHCs) and mESCs towards an early hepatic phenotype, by using five different proteins (collagen I, collagen III, collagen IV, laminin and fibronectin) in 32 combinations. This platform was used to identify specific ECM mixtures, containing either collagen I or fibronectin, that directed mESCs into a hepatic fate.

The differentiation profile of hESCs have also been examined on high-throughput biomaterial platforms [123,125,126]. The growth of hESCs on arrays with 18 different laminin-derived peptides was investigated by Derda *et al.* [125]. Their results revealed that the RNIAEIIKDI laminin peptide resulted in undifferentiated cells, while LGTIPG peptide promoted differentiation. Thus, they demonstrated that high-throughput platforms can be used to quickly identify peptide sequences that can regulate stem cell fate. In another study by Anderson *et al.* [123], the growth and differentiation of hESCs into cytokeratin positive cells on a microarray containing 1728 polymer mixtures were examined. Later, they investigated additional parameters such as root mean square roughness (0 –100 nm), stiffness (0.002 –2.262 GPa) and wettability (30–110°). They found that surface wettability and the elastic modulus of the polymers modulated the colony formation frequency (CFF) of hESCs, while surface roughness did not have a significant effect [126]. Taken together, these studies show that high-throughput screening platforms could more rapidly identify important parameters in culture dishes for better control of stem cell fate.

## 4. Conclusions

Surface topography as well as micro- and nanoscale chemical patterns on biomaterials have proven to be efficient methods to direct stem cell behavior. In addition, substrate stiffness and chemical cues are important factors in controlling stem cell fate. Further advances in controlling stem cell fate could be achieved by combining the above mentioned parameters in a more scalable and combinatorial manner to address the complexity of the natural stem cell niche. Overall, it is becoming clearer that the advances in micro- and nanoengineering can be used to precisely control stem cell behavior through cell-substrate interactions with enormous potential implications in science and medicine.
